# Development and validation of a web-based prediction tool on minor physical anomalies for schizophrenia

**DOI:** 10.1038/s41537-021-00198-5

**Published:** 2022-02-24

**Authors:** Xin-Yu Wang, Jin-Jia Lin, Ming-Kun Lu, Fong-Lin Jang, Huai-Hsuan Tseng, Po-See Chen, Po-Fan Chen, Wei-Hung Chang, Chih-Chun Huang, Ke-Ming Lu, Hung-Pin Tan, Sheng-Hsiang Lin

**Affiliations:** 1 Institute of Clinical Medicine, College of Medicine, National Cheng Kung University, Tainan, Taiwan; 2 Department of Psychiatry, Chi Mei Medical Center, Tainan, Taiwan; 3 Department of Health, Jianan Mental Hospital, Tainan, Taiwan; 4 Department of Applied Life Science and Health, Chia Nan University of Pharmacy and Science, Tainan, Taiwan; 5 Department of Psychiatry, National Cheng Kung University Hospital, College of Medicine, National Cheng Kung University, Tainan, Taiwan; 6 Department of Psychiatry, National Cheng Kung University Hospital, Dou-Liou Branch, Yunlin, Taiwan; 7 Institute of Behavioral Medicine, College of Medicine, National Cheng Kung University, Tainan, Taiwan; 8 Department of Obstetrics and Gynecology, National Cheng Kung University Hospital, College of Medicine, National Cheng Kung University, Tainan, Taiwan; 9 Department of Environmental and Occupational Health, College of Medicine, National Cheng Kung University, Tainan, Taiwan; 10 Department of Public Health, College of Medicine, National Cheng-Kung University, Tainan, Taiwan; 11 Biostatistics Consulting Center, National Cheng Kung University Hospital, College of Medicine, National Cheng Kung University, Tainan, Taiwan

**Keywords:** Biomarkers, Schizophrenia

## Abstract

In support of the neurodevelopmental model of schizophrenia, minor physical anomalies (MPAs) have been suggested as biomarkers and potential pathophysiological significance for schizophrenia. However, an integrated, clinically useful tool that used qualitative and quantitative MPAs to visualize and predict schizophrenia risk while characterizing the degree of importance of MPA items was lacking. We recruited a training set and a validation set, including 463 schizophrenia patients and 281 healthy controls to conduct logistic regression and the least absolute shrinkage and selection operator (Lasso) regression to select the best parameters of MPAs and constructed nomograms. Two nomograms were built to show the weights of these predictors. In the logistic regression model, 11 out of a total of 68 parameters were identified as the best MPA items for distinguishing between patients with schizophrenia and controls, including hair whorls, epicanthus, adherent ear lobes, high palate, furrowed tongue, hyperconvex fingernails, a large gap between first and second toes, skull height, nasal width, mouth width, and palate width. The Lasso regression model included the same variables of the logistic regression model, except for nasal width, and further included two items (interpupillary distance and soft ears) to assess the risk of schizophrenia. The results of the validation dataset verified the efficacy of the nomograms with the area under the curve 0.84 and 0.85 in the logistic regression model and lasso regression model, respectively. This study provides an easy-to-use tool based on validated risk models of schizophrenia and reflects a divergence in development between schizophrenia patients and healthy controls (https://www.szprediction.net/).

## Introduction

Minor physical anomalies (MPAs) are subtle morphological alterations in the head, eyes, ears, mouth, hands, and feet that do not have significant cosmetic or clinical effects^1^. Data from past research have proven that MPAs arise during the first or early second trimester and can be considered as indicators of altered brain development during early neurodevelopment due to schizophrenia^2–8^. Qualitative minor malformations represent defects of embryogenesis during the process of organogenesis, and the phenogenetic variation of final morphogenesis can be seen through quantitative defects^9^. Most of the current research on MPAs in schizophrenia has been based on qualitative observation, and only a few study groups have conducted quantitative measurements of MPAs^10^. Moreover, although MPAs have been suggested as biomarkers for schizophrenia, studies have rarely performed qualitative and quantitative measurements of MPAs in all six body parts (head, ears, eyes, mouth, hands, and feet) nor have they used a large number of cases for validation. Moreover, no research team has established systematic prediction tools for clinical use.

Extensive studies have shown that the MPA score of patients with schizophrenia is higher than that of healthy controls^5,11–20^. Some studies based on qualitative MPAs have reported that craniofacial anomalies are more often found in patients with schizophrenia than in healthy controls^21,22^ and seem to most accurately differentiate patients with schizophrenia from other patient groups^19,21^. Furthermore, according to a review paper, MPAs in the mouth and palate regions are more common than those in other anatomical sites. However, some studies have presented inconsistent results, with one showing that for five areas (namely mouth, feet, head, eyes, and ears) patients with schizophrenia have higher MPA scores than healthy controls^19^, but a systematic literature review reporting nobody region was more different or significant during MPA score testing than the others^12^. Reviewing the results of three papers that recorded detailed qualitative MPA items also did not lead to any consistent suggestions^23–25^. In two of the three studies, eight items exhibited significant differences between patients with schizophrenia and healthy controls (namely fine electric hair, hair whorls, epicanthus, low-seated ears, adherent ear lobes, steepled palate, furrowed tongue, and large gap between first and second toes), while six different items were significant in the last of the three studies (namely fused eyebrows, malformed ears, asymmetric ears, cuspidal ear, tongue with smooth-rough spots, and third toe longer than the second toe).

In previous quantitative assessments of MPAs, only a few studies on schizophrenia have reported comprehensive measurements covering multiple body areas, but rather, they have relied on observations of only specific parts, especially the palate and eye areas. From 2016 to 2020, four studies focused on the palate region. Two presented a significant difference in palate width between patients with schizophrenia and healthy controls^26,27^, and the other two reported differences in palate shape characteristics but did not find palate width to be significant^28,29^. Furthermore, a study measuring the quantitative characteristics of the eyes showed that patients with schizophrenia tended to have smaller optical angles and inner canthal distance. These results were supported by another study measuring the eye and skull regions^8^.

The objective of this study was to construct a web-based risk prediction tool for schizophrenia based on MPA measurements. The qualitative observations spanned six body areas and the detailed quantitative measurements covered five body areas. We used a large-scale training set to analyze and build the prediction model. A validation dataset was used to show the generalizability of the model, demonstrating the potential for clinical applications. Two different statistical methods, logistic regression, and Lasso regression were employed to establish nomograms for predicting the risk of schizophrenia. Comprehensive qualitative and detailed quantitative MPA measurements were assessed in patients with schizophrenia and healthy controls to identify the MPA measurements that could be used to distinguish patients with schizophrenia from healthy controls. Meanwhile, for clinical practice and research purposes, we developed a visualization and easy-to-use tool based on validated nomogram models that can predict the potential risk of schizophrenia.

## Results

### Descriptive data

Characteristics of patients from the training set (*n* = 500) and validation set (*n* = 244) are presented in Table 1. A comparison of the distribution of sex, age, body weight, height, and BMI between patients and healthy controls is presented. In the training set, patients had a mean age of onset of 25 years and a mean disease duration of 18 years. In the validation set, patients had a mean age of onset of 25 years and a mean disease duration of 17 years.

**Table 1:** Characteristics of participants in the training and validation sets.

Variables	Training set					Validation set				
	Schizophrenia patients		Healthy controls		*P*-value	Schizophrenia patients		Healthy controls		*P*-value
	(*n* = 320)		(*n* = 180)			(*n* = 143)		(*n* = 101)		
	*N*	%	*N*	%		*N*	%	*N*	%	
Male	197	61.0	79	43.8	<0.01	70	49.0	44	43.5	0.41
	Mean	SD	Mean	SD		Mean	SD	Mean	SD	
Age (year)	43	10.0	42	11.1	0.13	42	10.2	41	11.1	0.59
Weight (kg)	69	15.4	66	13.3	0.03	72	15.0	68	14.8	0.02
Height (cm)	165	8.6	164	8.0	0.58	164	8.1	164	7.9	0.61
BMI	25	4.9	24	4.1	0.06	27	4.9	26	4.9	0.02
Onset age (year)	25	7.9	0			25	7.8	0		
Disease duration (year)	18	9.8	0			17	9.7	0		

*BMI* body mass index, *SD* standard deviation.

### Nomogram variables identified based on logistic and Lasso regression

**Fig. 1:**
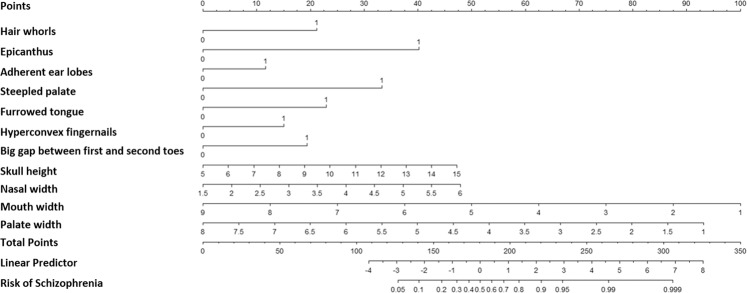
Nomogram of logistic regression analysis. A 11-variable nomogram established by logistic regression for predicting the risk of schizophrenia using minor physical anomalies.

**Fig. 2:**
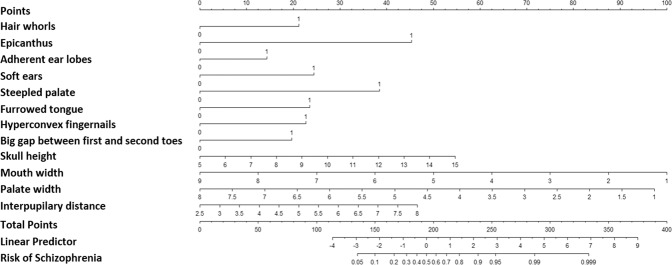
Nomogram of Lasso regression analysis. A 12-variable clinical nomogram established by Lasso regression for predicting the risk of schizophrenia using minor physical anomalies.

Variables in Table 2 were assessed through univariate and multivariate logistic regression analyses, which identified a total of 11 predictors, namely hair whorls, epicanthus, adherent ear lobes, high palate, furrowed tongue, hyperconvex fingernails, a large gap between first and second toes, skull height, nasal width, mouth width, and palate width as independent predictors of schizophrenia. These predictive factors were incorporated into a nomogram (Fig. 1). The Lasso regression analyses selected 12 variables to construct predictive nomogram, including hair whorls, furrowed tongue, skull height, interpupillary distance, mouth width, palate width, high palate, epicanthus, adherent ear lobes, soft ears, hyperconvex fingernails, and large gap between first and second toes (Fig. 2).

**Table 2:**
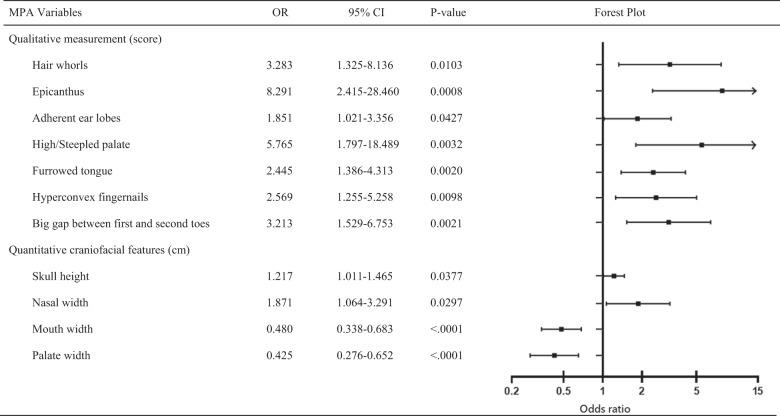
Results of a multivariable logistic regression model on MPA of schizophrenia.

Covariates with *P* < 0.05 in univariate analysis were entered in a multivariate logistic analysis model.

*MPA* minor physical anomalies, *OR* odds ratio.

To use the nomogram, the first variable result, i.e., a patient’s hair whorls, is located on the relevant axis. Next, a straight line is drawn upward from the value of the result to the point axis on the top of the nomogram to determine the points received based on hair whorls. This process is then repeated for every variable, and total points are calculated by adding all the points obtained from every variable. The final sum is located on the total points axis, and a straight line is drawn downward from there to obtain the risk of schizophrenia.

The logistic regression and Lasso regression models were implemented as an online risk prediction nomogram to calculate the expected risk of schizophrenia using qualitative and quantitative MPAs. This web-based tool is freely available at https://www.szprediction.net/, with all major browsers supported (Google Chrome, Internet Explorer, Mozilla Firefox, Safari, etc.). It contains the most useful items of MPAs to help clinical practice easily predict schizophrenia on an objective basis.

### Predicting the performance of the models

We have collected qualitative and quantitative MPAs data from the patients with schizophrenia and healthy controls and further divided these subjects into a training set and validation set to establish the nomogram predicting schizophrenia and executing the validation for the model.

**Fig. 3:**
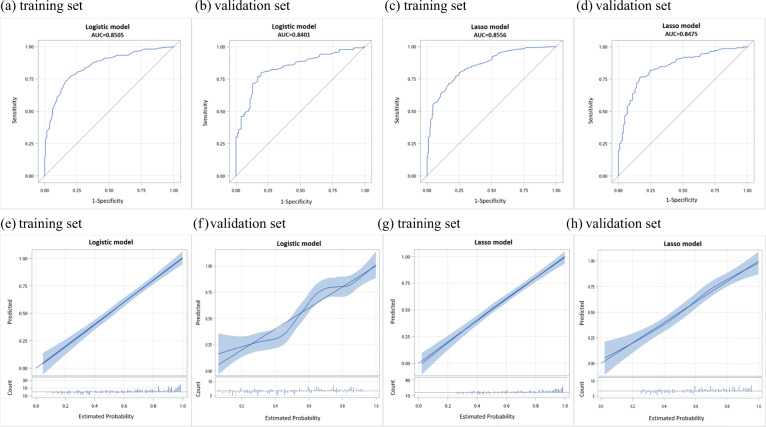
Receiver operating characteristic (ROC) curves and calibration curves for evaluating the discrimination performance of logistic regression and Lasso regression models in both training and validation cohorts. **a**–**d** ROC curves showing the capabilities of logistic regression and Lasso regression methods in predicting schizophrenia risk. **e**–**h** Calibration plot with a binary fringe plot of logistic regression and Lasso regression models.

In the logistic training set, the receiver operating characteristic (ROC) showed that the resulting model had quite good discrimination, with an area under the curve (AUC) of 0.85 (Fig. 3a). The logistic model also displayed good discrimination in the validation set, with the 0.84 AUC, 80.5% accuracy, 80.7% sensitivity, and 80.2% specificity (Fig. 3b). Of the 11 logistic regression model variables, 10 were included in the Lasso regression result, thus consolidating the model. The training set had an AUC of 0.85 and the validation set also showed good discrimination with the 0.85 AUC, 80.5% accuracy, 80.7% sensitivity, and 80.2% specificity (Fig. 3c, d). Furthermore, calibration plots of models were used to provide better information about the models, graphically showing good agreement between the predicted and observed data in the training and validation cohorts (Fig. 3e–h).

The sex, weight, and BMI showed significant differences between schizophrenia patients and healthy controls in the training set (Table 1). We also have tried to establish the MPAs nomogram with sex and BMI (Supplementary Figs. 1 and 2) in the logistic regression model (the AUCs of the training set and validation set were 0.86 and 0.84, respectively) and in the Lasso regression model (the AUCs of the training set and validation set were 0.87 and 0.84, respectively) (Supplementary Fig. 3). The above results were similar to the results of the model without sex and BMI. In addition, we stratified the male and female subjects in the training set to perform the ROC curve analysis by both logistic regression analysis and lasso regression analysis, respectively. We further use the validation dataset to analyze the performance of models by ROC curve analysis. In male-based models, the AUC was 0.81 and 0.89 in the logistic and lasso regression model, respectively (Supplementary Fig. 4). In female-based models, the AUC was 0.87 and 0.91 in the logistic and lasso regression model, respectively (Supplementary Fig. 4).

## Discussion

To the best of our knowledge, this study on the measurement of MPAs in patients with schizophrenia enrolled the largest number of participants of Chinese origin. The reported prediction models predict the risk of schizophrenia by utilizing a nomogram, which was established through logistic or Lasso regression and is readily available. In addition, we measured qualitative and quantitative individual items across six regions of the body to investigate the items that had the greatest predictive power. The most important contribution of this study is that it provides a web-based prediction tool for clinical use. This tool has been tested on hundreds of cases to verify the reliability of the models.

Studies have indicated that MPAs are a reliable marker for distinguishing patients with schizophrenia from healthy controls^7^. However, the items that are most useful among MPAs and should be used to predict schizophrenia remain unclear. Eleven items of MPAs were included as predictors of schizophrenia based on the logistic regression model, and 12 items were adopted in the Lasso regression model. The Lasso regression model included all the items of the logistic regression model except for nasal width. Both the logistic and the lasso regression models achieved good performance in the training and validation sets with high AUC values, suggesting the reliability of both the models for the classification of schizophrenia. Our improved models achieved higher predictive efficacy for schizophrenia than those reported in previous studies, which correctly classified 81.3 and 84.5% of cases^13,23^.

There were many studies that used logistic regression or lasso regression to build nomograms^30–32^. Several studies also used logistic regression to determine which variables of MPAs can discriminant schizophrenia patients from healthy controls^10,33,34^. Lasso regression is a shrinkage and variable selection method for regression models to avoid overfitting the data in the analysis and overestimation of how well the model performs^35,36^. Since lasso regression has the advantages that can reducing the complexity of high dimensional data, we used this method to confirm the stability of the model. In addition, these two statistical methods do not remove the need to validate a model in an external dataset. Therefore, the models of this study should undertake more validation in the future. In the study, we considered that logistic and lasso regression models are suitable for selecting MPA variables to discriminate schizophrenia patients from healthy controls, so the two methods were used to build the nomogram models.

Studies have shown that hair variations are related to brain anomalies because they have a common developmental origin^22,25^. Our results showed that people with ≥2 hair whorls are more likely to be patients, and this is in accordance with the results of a study that measured MPAs in a cohort with schizophrenia within the Serbian population; this study reported that approximately 65.1% of patients with schizophrenia had ≥2 hair whorls^25^. Our results showed that the epicanthus is part of the proposed checklist and this has been proven significant in several previous studies^13,23,24,37,38^. However, the fact that the epicanthus is more common in the Asian population suggests that MPAs differ with ethnic differences between Asian and other populations^24,37^. The study participants were homogeneous (all Chinese) and therefore the results do not apply to other ancestral populations. Adherent ear lobes were significant in our study and in two previous studies^23,24^. Moreover, this qualitative feature is unlikely to be influenced by age^5^. The palate is believed to be a typical structure in terms of schizophrenia-related MPAs, which originates at 6–9 weeks of pregnancy and continues to develop until 16–17 weeks. Palatal differences in patients with schizophrenia are noteworthy findings. The formation of the primary palate involves a series of local cellular changes during the craniofacial growth period^39^. Our results revealed that patients with schizophrenia have a significantly higher and narrower palate. This finding is consistent with those of most studies^3,4,21,40^, but failed to agree with those presented in a paper published in 2016; that study reported that patients with schizophrenia have a significantly wider palate^26^. The inclusion of the high palate and furrowed tongue in both the models shows that our results are consistent with the findings that palate and tongue anomalies represent the highest prevalence of MPAs in the mouth region of patients with schizophrenia^8,25,41,42^. Our results revealed that patients with schizophrenia have a greater skull height and nasal width, and smaller mouth width and palate width.

Moreover, the result of a previous study showed schizophrenia patients were significantly more likely to have MPA than normal controls in both genders, but the difference was tended to be more pronounced in males^38^. This may be related to the fact that the male fetus is more vulnerable to injury during prenatal development^43^. Previous studies also pointed out that males have lower resistance in the processes of brain development^44,45^. However, considering the influence of gender on the risk models in this study. We added sex weight and BMI into the models, and the results were similar to the results of the model without sex and BMI. We do not exclude that gender may have an impact on risk prediction, but in this study, gender seems to have little effect on the prediction accuracy of our models. In addition, we also selected MPA variables for the risk of schizophrenia according to different genders. The results showed that high palate and palate width were included in both male and female logistic and lasso regression models, which may imply that the characteristics of the palate were suitable for both men and women to assess the risk of schizophrenia.

This study has the following limitations. First, we performed linear measurements, and this may not be able to completely evaluate three-dimensional structures, for example, that of the palate. Second, we did not assess the subtypes of schizophrenia because schizophrenia has many different subtypes and these may be associated with different symptoms or require different treatments. Whether a specific subtype of schizophrenia has certain MPA features still needs to be determined. Third, our participants were all of Taiwanese Han Chinese origin, and patients of different ethnicities are required to further validate our prediction model. Fourth, we only investigated the relationship between MPAs and schizophrenia in this study; moreover, the relationships between MPAs and other psychiatric disorders need more research in the future.

In conclusion, this study on the measurement of MPAs in patients with schizophrenia had the largest number of participants of Chinese origin. Our findings support the hypothesis of abnormal neurodevelopment in schizophrenia and may provide more understanding of the pathophysiology of schizophrenia. Furthermore, this is the study using MPAs to establish a pioneering web-based tool based on validated risk models of schizophrenia for clinical practice or research purposes. Our easy-to-use nomograms were not intended to replace standard diagnostic methods, but to help researchers or clinicians better assess the risk of schizophrenia. Additional replications of the topic are warranted to test the reported outcomes.

## Methods

### Study subjects

The present study was conducted using data taken from 463 patients of Taiwanese Han Chinese origin with schizophrenia who were recruited from five medical institutions in southern Taiwan: Chi Mei Medical Center, Jianan Mental Hospital, Lok An Hospital, National Cheng Kung University Hospital, and National Cheng Kung University Hospital Dou-Liou Branch. All patients were diagnosed as having schizophrenia by professional psychiatrists of the participating hospitals based on the *Diagnostic and Statistical Manual of Mental Disorders*, DSM-IV-TR (2011–2018) or DSM-5 (2018–2021). For comparison, 281 people without a history of psychiatric disorders were recruited from the hospital staff and community and allocated to the healthy control group. The data collected included the participants’ baseline descriptions and their measured MPAs, which could be utilized as indicators of underlying disease susceptibility. The study excluded subjects with histories of illegal substance or alcohol abuse, identifiable neurological disorders, clinical mental retardation, somatic disorders with neurological components, or those whose parents were not Han Chinese. The study design and recruitment procedures received ethical approval from the institutional review boards (IRBs) of the participating hospitals (IRB numbers: 10102-006, 11-011, B-BR-103-036-T, 10301-002, 10612-011, B-BR-106-088, B-BR-108-094, and 10901-006). Written informed consent was obtained from the participants.

### Measurements of MPAs and craniofacial features

The qualitative and quantitative assessments of MPAs have performed the Waldrop scale and other scales based on resources^34^. Using the combined standardized MPA scale in six body regions: head, ears, eyes, mouth, hands, and feet; 41 qualitative assessments and 27 quantitative measurements were performed.

We developed qualitative MPAs using a manual scoring system, with 16 qualitative measurements and 23 new items based on the Waldrop scale^46^ and the study by Ismail et al.^1^. We further added two measurement items, strabismus, and cuspidal ear, into the qualitative assessment of MPAs based on a Japanese study^37^. In the qualitative assessment, 33 items acted as binary variants. We assigned normal present variants a score of 0 and absent or other variants a score of 1. The other 8 qualitative assessment items were scored using an ordinal scale (0–2) to represent the magnitude of the anomaly. MPAs were assessed separately on the right and left sides in 28 bilateral anatomical sites. A total qualitative MPA score was calculated by adding all the scores from each region, with a range between 0 and 83.

The quantitative MPAs consisted of 27 items from the scales of Lane et al.^4^, McGrath et al.^11^, and Elizarraras-Rivas et al.^47^, which included separate right- and left-side measurements taken at 11 bilateral anatomical sites. It also included two items (head circumference and canthal distance) from the Waldrop scale, and we further divided canthal distance into inner and outer canthal distances. The quantitative measurements were conducted using standardized methods with the two most-cited books on anthropometric measurements used as references^48,49^.

Two well-trained research assistants collected all qualitative and craniofacial measurement data from 20 healthy subjects. The range of inter-rater reliability for the qualitative items was 0.95–1.00, whereas the range of intraclass correlation coefficients for the quantitative items was 0.70–0.96.

### Statistical analysis

Demographic parameters including sex, education level, weight, height, and body mass index (BMI) were evaluated. Continuous variables are expressed as the mean and standard deviation, and categorical variables are presented as frequencies and proportions. Between‐group comparisons, Student’s two-sided *t*-test was used for continuous variables and Pearson’s chi-square test was used for categorical variables. The nomogram was constructed using the following modeling process. We first assigned the patients of three hospitals (Jianan Mental Hospital, Lok An Hospital, and National Cheng Kung University Hospital) to a training set and the patients of the other two hospitals (Chi Mei Medical Center and National Taiwan University Hospital Yun-Lin Branch) to a validation set. A total of 320 patients with schizophrenia were assigned to the training set and 143 were assigned to the validation set. To match the ratio of cases and controls, we randomly assigned two-thirds of the control group (*n* = 180) to the training set and one-third of the control group (*n* = 101) to the validation set.

In order to simplify the complex scoring, we combine the left and right features of the qualitative items. As long as there were variants on the left or right sides, the score would be assigned as 1. The measurements were averaged on the left and right sides of the quantitative items. Logistic regression analyses were applied to present significant variables. A total of 68 variables were first analyzed by univariate analysis, then there were 24 variables that got a *p*-value < 0.05. We further analyzed these 24 variables by multivariate analysis. Finally, based on the results of the multivariate model, there were 11 predictors were got a *p*-value < 0.05 and were used to construct nomograms for predicting the risk of schizophrenia. Lasso regression was used to select variables as a double-check for logistic regression results. Lasso regression can force certain coefficients to be set to zero, effectively selecting a simpler model that does not contain those coefficients. The optimal value for the tuning parameter *λ* within one standard error of the minimum was determined with cross-validation (Supplementary Fig. 5). A total of 68 variables were first analyzed by Lasso regression, then 16 variables with *p*-value < 0.05 were included in the multivariate analysis. Finally, 12 predictors with a *p*-value < 0.05 were used to construct prediction nomograms of schizophrenia.

The prediction model formed from the training set was applied to the validation set to validate and evaluate the prediction efficacy. We plotted the ROC curve and calculated the AUC to verify the discrimination performance in both the training and validation sets. The AUC represents the model’s ability to discriminate between patients with schizophrenia and those without. AUC must be between 0 and 1. An AUC of 1 denotes that both sensitivity and specificity are 100%, indicating perfect concordance, whereas an AUC of 0.5 indicates a result equal to chance. The nomograms were further evaluated to determine sensitivity and specificity. High sensitivity and specificity values confirm that the ability of the nomogram to accurately identify schizophrenia is good. A calibration plot with a binary fringe plot was generated for the accuracy of the nomogram, which compared predicted probabilities with the observed incidence of schizophrenia. If the smoother lies close to the diagonal, the model is well-calibrated. The butterfly fringe plot is shown in a panel below the predicted probabilities. It indicates the counts of the responses by using lines of various lengths. Lines that point downward indicate the number of counts for *Y* = 0, whereas lines that point upward indicate the number of counts for *Y* = 1. All analyses were performed using SAS 9.4 (SAS Institute Inc., Cary, NC, USA) and R Studio (version 1.2.1335, R Foundation, Vienna, Austria).

### Reporting summary

Further information on research design is available in the Nature Research Reporting Summary linked to this article.

## Supplementary information

Supplementary figure

REPORTING SUMMARY

## Data Availability

Due to ethical restrictions and conditions on participant consent, the datasets in the current study are not publicly available.

## Code availability

The SAS code of the study findings is available from the corresponding author upon reasonable request.

## References

[1] Ismail B, Cantor-Graae E, McNeil TF (1998). Minor physical anomalies in schizophrenic patients and their siblings. Am. J. Psychiatry.

[2] Buckley PF (1998). The clinical stigmata of aberrant neurodevelopment in schizophrenia. J. Nerv. Ment. Dis..

[3] Green MF, Satz P, Gaier DJ, Ganzell S, Kharabi F (1989). Minor physical anomalies in schizophrenia. Schizophr. Bull..

[4] Lane A (1997). The anthropometric assessment of dysmorphic features in schizophrenia as an index of its developmental origins. Psychol. Med..

[5] Compton MT, Walker EF (2009). Physical manifestations of neurodevelopmental disruption: are minor physical anomalies part of the syndrome of schizophrenia?. Schizophr. Bull..

[6] McNeil TF, Cantor‐Graae E (2000). Minor physical anomalies and obstetric complications in schizophrenia. Aust. N. Z. J. Psychiatry.

[7] Sivkov ST, Akabaliev VH (2004). Discriminating value of total minor physical anomaly score on the Waldrop physical anomaly scale between schizophrenia patients and normal control subjects. Schizophr. Bull..

[8] Wang Y (2016). A trend toward smaller optical angles and medial‐ocular distance in schizophrenia spectrum, but not in bipolar and major depressive disorders. Psych. J..

[9] Hajnal A (2016). Minor physical anomalies are more common among the first-degree unaffected relatives of schizophrenia patients—results with the Méhes Scale. Psychiatry Res..

[10] Tsai I-N (2016). Improving risk assessment and familial aggregation of age at onset in schizophrenia using minor physical anomalies and craniofacial measures. Medicine.

[11] McGrath J (2002). Minor physical anomalies and quantitative measures of the head and face in patients with psychosis. Arch. Gen. Psychiatry.

[12] Weinberg SM, Jenkins EA, Marazita ML, Maher BS (2007). Minor physical anomalies in schizophrenia: a meta-analysis. Schizophr. Res..

[13] Huang C-J (2010). Significance of morphological features in schizophrenia of a Chinese population. J. Psychiatr. Res..

[14] Aksoy-Poyraz C, Poyraz BÇ, Turan Ş, Arıkan MK (2011). Minor physical anomalies and neurological soft signs in patients with schizophrenia and their siblings. Psychiatry Res..

[15] Compton MT, Chan RC, Walker EF, Buckley PF (2011). Minor physical anomalies: potentially informative vestiges of fetal developmental disruptions in schizophrenia. Int. J. Dev. Neurosci..

[16] Xu T, Chan RC, Compton MT (2011). Minor physical anomalies in patients with schizophrenia, unaffected first-degree relatives, and healthy controls: a meta-analysis. PloS ONE.

[17] Golembo-Smith S (2012). Premorbid multivariate markers of neurodevelopmental instability in the prediction of adult schizophrenia-spectrum disorder: a high-risk prospective investigation. Schizophr. Res..

[18] Gassab L, Aissi M, Slama H, Gaha L, Mechri A (2013). Prevalence and score of minor physical anomalies in patients with schizophrenia and their first degree relatives: a Tunisian study. Compr. Psychiatry.

[19] Akabaliev VH, Sivkov ST, Mantarkov MY (2014). Minor physical anomalies in schizophrenia and bipolar I disorder and the neurodevelopmental continuum of psychosis. Bipolar Disord..

[20] Tényi T (2015). Minor physical anomalies are more common in schizophrenia patients with the history of homicide. Psychiatry Res..

[21] Waddington JL (1999). Early cerebro-craniofacial dysmorphogenesis in schizophrenia: a lifetime trajectory model from neurodevelopmental basis to ‘neuroprogressive’process. J. Psychiatr. Res..

[22] Gourion D (2004). Minor physical anomalies in patients with schizophrenia and their parents: prevalence and pattern of craniofacial abnormalities. Psychiatry Res..

[23] Akabaliev V, Sivkov S, Mantarkov M, Ahmed-Popova F (2011). Biomarker profile of minor physical anomalies in schizophrenia patients. Folia Med..

[24] Lin Y (2012). Minor physical anomalies in patients with schizophrenia in a Chinese population. Psychiatry Res..

[25] Babović SS (2019). Craniofacial measures and minor physical anomalies in patients with schizophrenia in a cohort of Serbian population. Srp. Ark. Celok. Lek..

[26] Delice M, Gurbuz O, Oflezer C, Kurt E, Mandali G (2016). Palate size and shape in schizophrenia. Psychiatry Res..

[27] Kirkpatrick B, Gürbüz Oflezer Ö, Delice Arslan M, Hack G, Fernandez-Egea E (2019). An early developmental marker of deficit versus nondeficit schizophrenia. Schizophr. Bull..

[28] Franco RPAV (2019). Morphology of the palate, palatal rugae pattern, and dental arch form in patients with schizophrenia. Spec. Care Dent..

[29] Mobile RZ (2020). The characteristics of palate and upper dental arch can be an anatomical marker for men with schizophrenia? Case-control study: palate can be a marker for schizophrenia?. Spec. Care. Dent..

[30] Wang S, Tu J, Dong K (2019). Nomogram to predict postoperative PR in patients undergoing CT-guided transthoracic lung biopsy. J. Thorac. Dis..

[31] Sha L (2021). Predictors of death within 6 months of stroke onset: A model with Barthel index, platelet/lymphocyte ratio and serum albumin. Nurs. Open.

[32] Liu H (2019). Derivation and validation of a nomogram to predict in‐hospital complications in children with tetralogy of fallot repaired at an older age. J. Am. Heart Assoc..

[33] Iasevoli F (2018). Clinical evaluation of functional capacity in treatment resistant schizophrenia patients: comparison and differences with non-resistant schizophrenia patients. Schizophr. Res..

[34] Lin A-S (2015). Minor physical anomalies and craniofacial measures in patients with treatment-resistant schizophrenia. Psychol. Med..

[35] Hepp T, Schmid M, Gefeller O, Waldmann E, Mayr A (2016). Approaches to regularized regression–a comparison between gradient boosting and the lasso. Methods Inf. Med..

[36] Ranstam J, Cook J (2018). LASSO regression. Br. J. Surg..

[37] Yoshitsugu K (2006). A novel scale including strabismus and ‘cuspidal ear’for distinguishing schizophrenia patients from controls using minor physical anomalies. Psychiatry Res..

[38] Akabaliev VH, Sivkov ST (2003). Sexual dimorphism in minor physical anomalies in schizophrenic patients and normal controls. Compr. Psychiatry.

[39] Diewert VM, Wang K-Y (1992). Recent advances in primary palate and midface morphogenesis research. Crit. Rev. Oral. Biol. Med..

[40] Guy JD, Majorski LV, Wallace CJ, Guy MP (1983). The incidence of minor physical anomalies in adult male schizophrenics. Schizophr. Bull..

[41] Jaaro-Peled H, Sawa A (2020). Neurodevelopmental factors in schizophrenia. Psychiatr. Clin..

[42] Trixler M, Tenyi T, Csábi G, Szabo R (2001). Minor physical anomalies in schizophrenia and bipolar affective disorder. Schizophr. Res..

[43] Lewis S (1992). Sex and schizophrenia: vive la difference. Br. J. Psychiatry.

[44] Castle DJ, Murray RM (1991). The neurodevelopmental basis of sex differences in schizophrenia1. Psychol. Med..

[45] O’Callaghan, E., Larkin, C., Kinsella, A. & Waddington, J. L. Familial, obstetric, and other clinical correlates of minor physical anomalies in schizophrenia. *Am. J. Psychiatry* (1991). 10.1176/ajp.148.4.479 2006694

[46] Waldrop MF, Pedersen FA, Bell RQ (1968). Minor physical anomalies and behavior in preschool children. Child Dev..

[47] Elizarraras-Rivas J, Fragoso-Herrera R, Cerdan-Sanchez L, Ramos-Zepeda R, Barajas-Barajas L (2003). Minor physical anomalies and anthropometric measures in schizophrenia: a pilot study from Mexico. Schizophr. Res..

[48] Judith, G., Froster-Iskenius, U. G. & Allanson, J. E. *Handbook of Normal Physical Measurements*. (Oxford University Press, 1989).

[49] Farkas, L. G. *Anthropometry of the Head and Face*. (Lippincott Williams & Wilkins, 1994).

