# Supplementary material for: Development and validation of a web-based prediction tool on minor physical anomalies for schizophrenia
Source: Schizophrenia (Heidelb). 2022 Feb 24;8(1):4. doi: 10.1038/s41537-021-00198-5 (PMC8873231; doi:10.1038/s41537-021-00198-5)
Supplement: Supplementary file 1 — Supplementary figure [file 41537_2021_198_MOESM1_ESM.pdf]

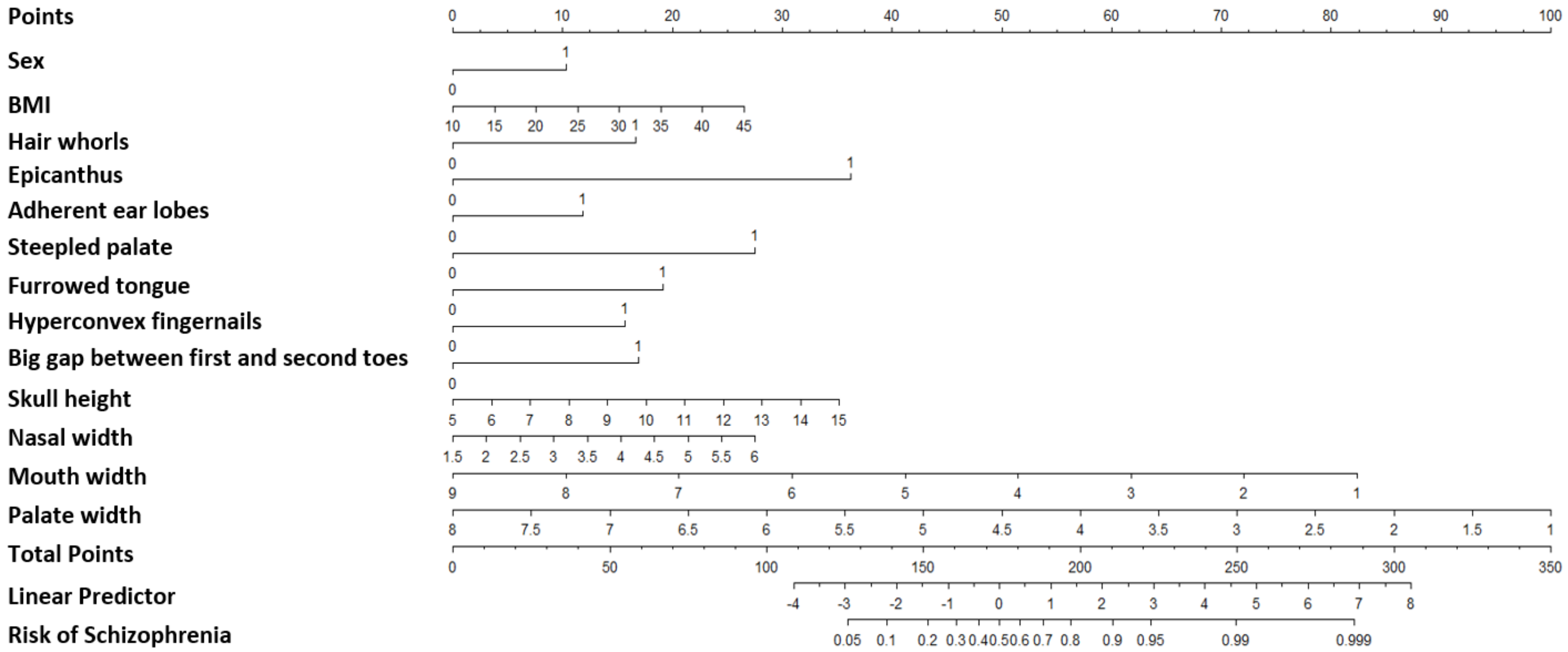

Supplementary Fig. 1 Nomogram with sex and BMI for predicting the risk of schizophrenia using minor physical anomalies based on logistic regression.

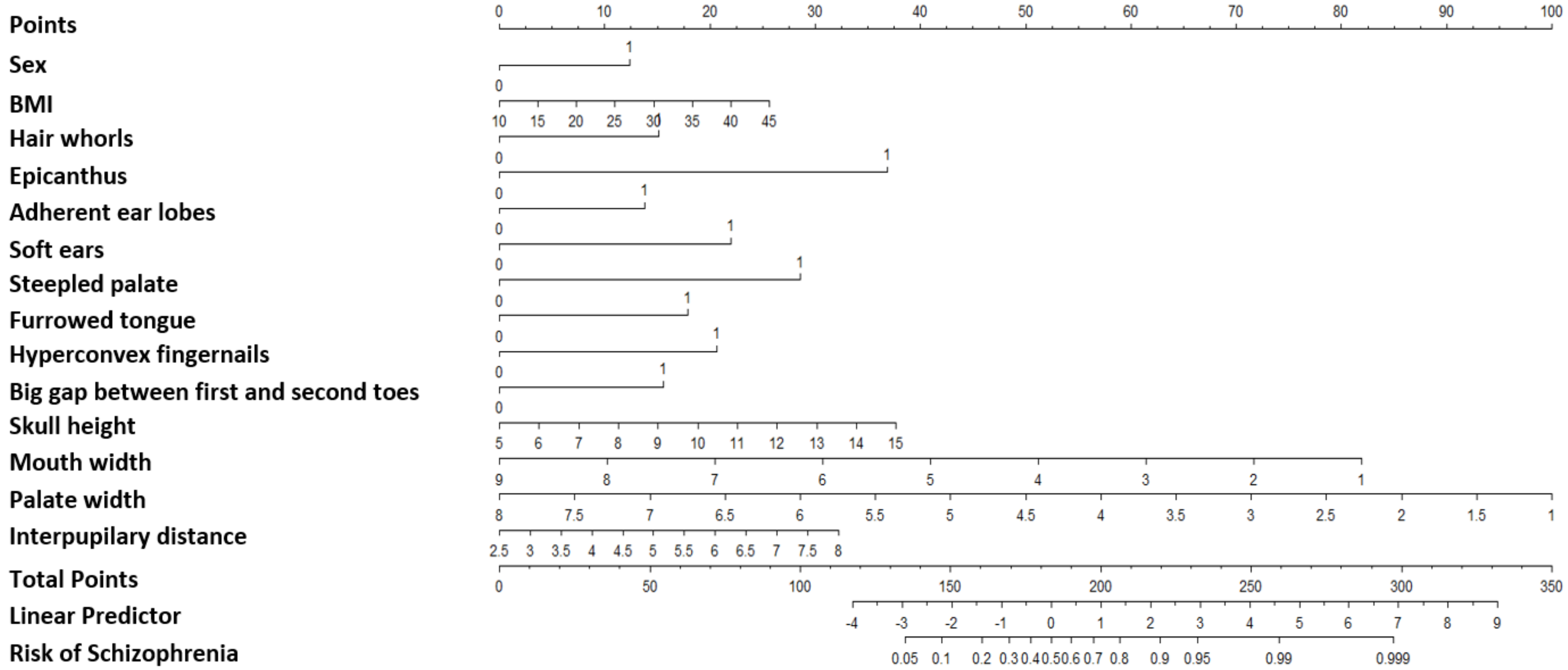

Supplementary Fig. 2 Nomogram with sex and BMI for predicting the risk of schizophrenia using minor physical anomalies based on lasso regression.

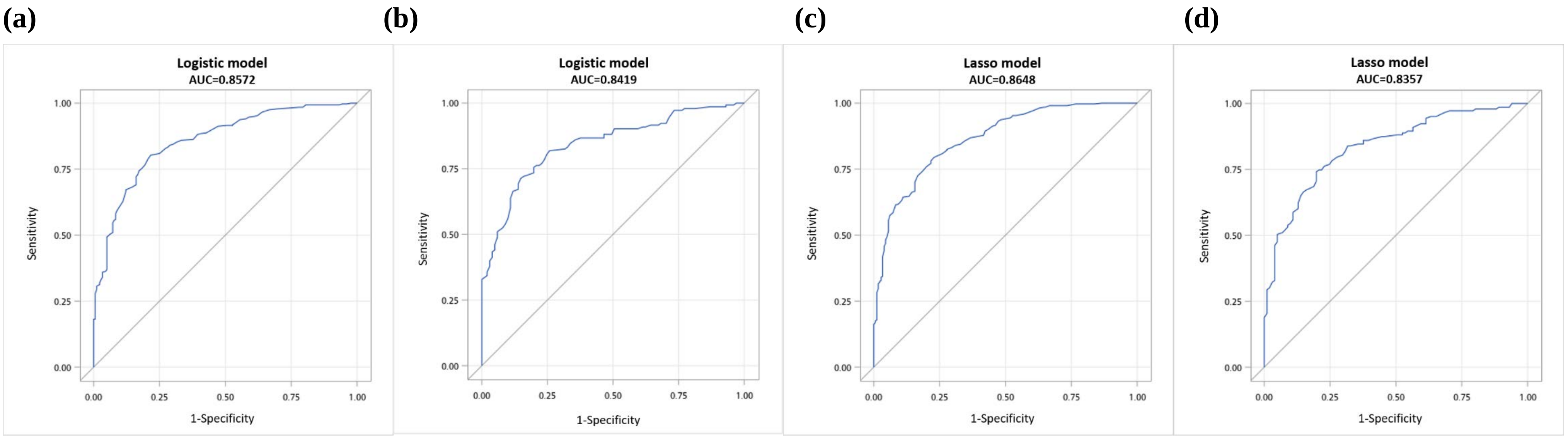

Supplementary Fig. 3

(a) (b) ROC curves for the logistic regression model with sex and BMI in training and validation sets; area under the curves (AUCs) were 0.8572 and 0.8419, respectively.

(c) (d) ROC curves for the lasso regression model with sex and BMI in training and validation sets; AUCs were 0.8648 and 0.8357, respectively.

(a)

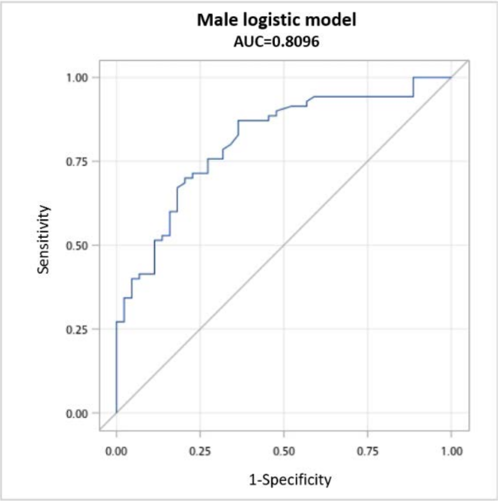

(b)

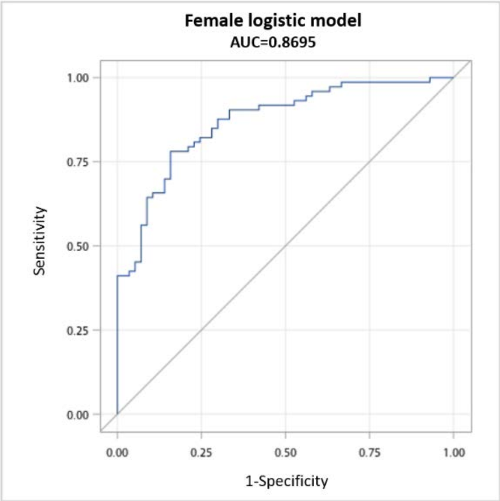

(c)

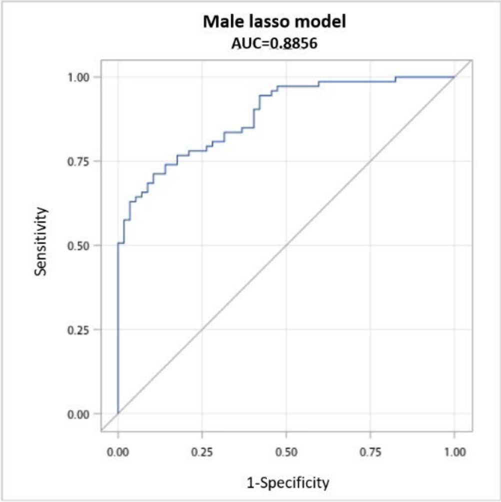

(d)

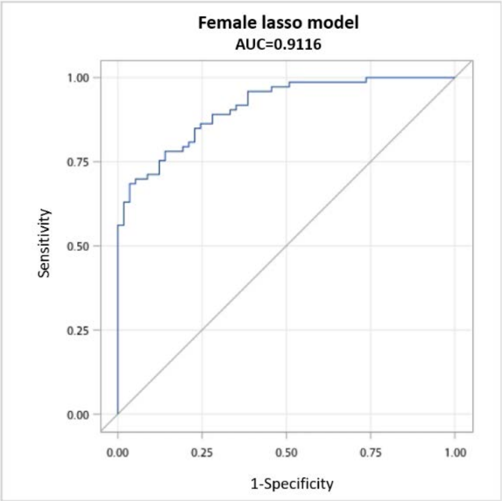

Supplementary Fig. 4

(a) (b) ROC curves for the male and female logistic regression models in validation sets, the area under the curves (AUCs) were 0.8096 and 0.8695, respectively.

(c) (d) ROC curves for the male and female the lasso regression models in validation sets, the AUCs were 0.8856 and 0.9116, respectively.

(a)

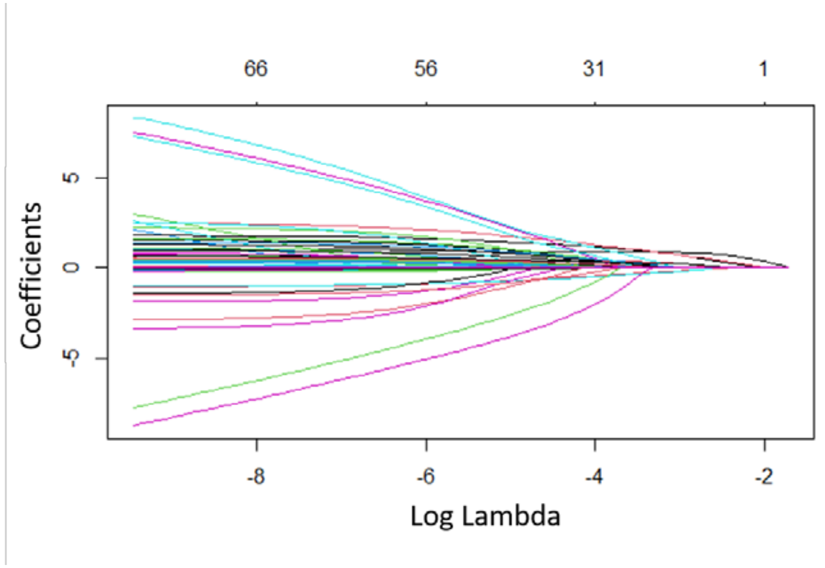

(b)

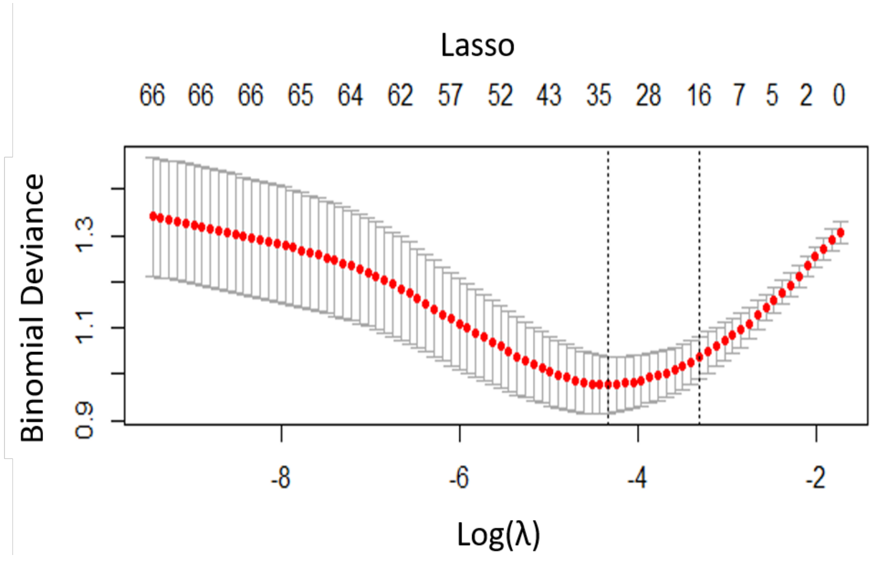

Supplementary Fig. 5 Predictors selected using lasso regression.

(a) Lasso coefficient profiles of all minor physical anomaly (MPA) features.

(b) Identification of the optimal penalization coefficient λ in the lasso model with 10-fold cross-validation and the one standard error criterion.

The first plot (left) shows the predictor coefficient scores as a function of log(λ), indicating the shrinkage of coefficients for larger values of log(λ). The top numbering of the plot indicates the number of variables the model is using, going from all predictors (top left corner) to sparser models (top right corner). The second plot (right) demonstrates the 10-fold cross-validated mean squared error as a function of log(λ) for the lasso regularized model using the full data with interaction terms. The top numbering of the plot indicates the number of variables the model is using, going from all predictors (top left corner) to sparser models (top right corner). This function helps the optimization of lasso in terms of choosing the best λ.
