# Supplementary material for: Development and validation of a web-based prediction tool on minor physical anomalies for schizophrenia
Source: Schizophrenia (Heidelb). 2022 Feb 24;8(1):4. doi: 10.1038/s41537-021-00198-5 (PMC8873231; doi:10.1038/s41537-021-00198-5)
Supplement: Supplementary file 2 — REPORTING SUMMARY [file 41537_2021_198_MOESM2_ESM.pdf]

nature portfolio

Corresponding author(s): Dr Sheng-Hsiang Lin

Last updated by author(s): Nov 16, 2021

Reporting Summary

Nature Portfolio wishes to improve the reproducibility of the work that we publish. This form provides structure for consistency and transparency in reporting. For further information on Nature Portfolio policies, see our Editorial Policies and the Editorial Policy Checklist.

Statistics

For all statistical analyses, confirm that the following items are present in the figure legend, table legend, main text, or Methods section.

n/a	Confirmed	
☐	☒	The exact sample size (n) for each experimental group/condition, given as a discrete number and unit of measurement
☐	☒	A statement on whether measurements were taken from distinct samples or whether the same sample was measured repeatedly
☐	☒	The statistical test(s) used AND whether they are one- or two-sided<br>*Only common tests should be described solely by name; describe more complex techniques in the Methods section.*
☐	☒	A description of all covariates tested
☐	☒	A description of any assumptions or corrections, such as tests of normality and adjustment for multiple comparisons
☐	☒	A full description of the statistical parameters including central tendency (e.g. means) or other basic estimates (e.g. regression coefficient) AND variation (e.g. standard deviation) or associated estimates of uncertainty (e.g. confidence intervals)
☐	☒	For null hypothesis testing, the test statistic (e.g. F, t, r) with confidence intervals, effect sizes, degrees of freedom and P value noted<br>*Give P values as exact values whenever suitable.*
☒	☐	For Bayesian analysis, information on the choice of priors and Markov chain Monte Carlo settings
☒	☐	For hierarchical and complex designs, identification of the appropriate level for tests and full reporting of outcomes
☐	☒	Estimates of effect sizes (e.g. Cohen's d, Pearson's r), indicating how they were calculated

Our web collection on statistics for biologists contains articles on many of the points above.

Software and code

Policy information about availability of computer code

Data collection	no software was used.
Data analysis	The SAS code that supports the findings of this study are available from the corresponding author upon reasonable request.

For manuscripts utilizing custom algorithms or software that are central to the research but not yet described in published literature, software must be made available to editors and reviewers. We strongly encourage code deposition in a community repository (e.g. GitHub). See the Nature Portfolio guidelines for submitting code & software for further information.

Data

Policy information about availability of data

All manuscripts must include a data availability statement. This statement should provide the following information, where applicable:

- Accession codes, unique identifiers, or web links for publicly available datasets
- A description of any restrictions on data availability
- For clinical datasets or third party data, please ensure that the statement adheres to our policy

The datasets used and analysed in the current study are not publicly available due to conditions on participant consent and other ethical restrictions

Field-specific reporting

Please select the one below that is the best fit for your research. If you are not sure, read the appropriate sections before making your selection.

☒ Life sciences ☐ Behavioural & social sciences ☐ Ecological, evolutionary & environmental sciences

For a reference copy of the document with all sections, see nature.com/documents/nr-reporting-summary-flat.pdf

Life sciences study design

All studies must disclose on these points even when the disclosure is negative.

Sample size	The power is more than 0.8 based on the sample size of the study.
Data exclusions	The study excluded subjects with histories of illegal substance or alcohol abuse, identifiable neurological disorders, clinical mental retardation, somatic disorders with neurological components, or those whose parents were not Han Chinese.
Replication	The range of inter-rater reliability for the qualitative items was 0.95–1.00, whereas the range of intraclass correlation coefficients for the quantitative items was 0.70–0.96. The logistic model displayed good discrimination in the validation set, with the 0.84 AUC, 80.5% accuracy, 80.7% sensitivity, and 80.2% specificity. The lasso model also showed good discrimination in the validation set, with the 0.85 AUC, 80.5% accuracy, 80.7% sensitivity, and 80.2% specificity.
Randomization	To match the ratio of cases and controls, we randomly assigned two-thirds of the control group (n = 180) to the training set and one-third of the control group (n = 101) to the validation set.
Blinding	This study is not blind because of medical needs of the patients.

Reporting for specific materials, systems and methods

We require information from authors about some types of materials, experimental systems and methods used in many studies. Here, indicate whether each material, system or method listed is relevant to your study. If you are not sure if a list item applies to your research, read the appropriate section before selecting a response.

Materials & experimental systems

n/a	Involved in the study	
☒	☐	Antibodies
☒	☐	Eukaryotic cell lines
☒	☐	Palaeontology and archaeology
☒	☐	Animals and other organisms
☐	☒	Human research participants
☒	☐	Clinical data
☒	☐	Dual use research of concern

Methods

n/a	Involved in the study	
☒	☐	ChIP-seq
☒	☐	Flow cytometry
☒	☐	MRI-based neuroimaging

Human research participants

Policy information about studies involving human research participants

Population characteristics	The demographic and clinical profiles of the participants are minor physical anomalies, gender, age, weight, height, BMI, onset age, and disease duration.
Recruitment	The present study was conducted using data taken from 463 patients of Taiwanese Han Chinese origin with schizophrenia who were recruited from five medical institutions in southern Taiwan: Chi Mei Medical Center, Jianan Mental Hospital, Lok An Hospital, National Cheng Kung University Hospital, and National Taiwan University Hospital Yun-Lin Branch. For comparison, 281 people without a history of psychiatric disorders were recruited from the hospital staff and community and allocated to the healthy control group.
Ethics oversight	The study design and recruitment procedures received ethical approval from the institutional review boards (IRBs) of the participating hospitals (IRB numbers: 10102-006, 11-011, B-BR-103-036-T, 10301-002, 10612-011, B-BR-106-088, B-BR-108-094 and 10901-006). Written informed consent was obtained from the participants.

Note that full information on the approval of the study protocol must also be provided in the manuscript.
